# “Football- It’s in Your Blood”—Lived Experiences of Undertaking Recreational Football for Health in Older Adults

**DOI:** 10.3390/ijerph192214816

**Published:** 2022-11-10

**Authors:** Sophie Mowle, Emma Eyre, Mark Noon, Jason Tallis, Michael J. Duncan

**Affiliations:** Centre for Sport, Exercise, and Life Sciences, Coventry University, Coventry CV1 5FB, UK

**Keywords:** walking football, older adults, physical activity, health, behaviour change theory

## Abstract

Physical inactivity is prevalent in older adults and contributes to age-related decline in function, health, well-being, and quality of life. Recreational football for older adults has shown promise for promoting health benefits. This study explores the lived experiences of older adults engaging in a walking and recreational football intervention and identifies factors that affect behaviours and can encourage change in this population. A purposive sample (n = 14; aged 67 ± 5 years) of the lived experiences of those participating in a recreational football intervention took part in two focus groups. The participants’ responses were grouped into three-time reflecting specific points in their lives: what stopped them from playing football, what got them playing, and what is needed for them to continue playing in the future. Within each of these time points in their lives, themes were identified. The key findings and practical recommendations were that football needs to be adapted and local, that the priority to play football changes over time, and that football itself is a fundamentally intrinsic motivator; ‘it’s in your blood’. The findings can be used to inform future interventions, encourage participation, and advise on the best practices for key stakeholders in the physical activity domain.

## 1. Introduction

Due to its importance in enhancing health, well-being, and physical function, physical activity is considered a key behaviour for older adults to undertake [1]. Evidence has shown that being physically active can reduce the risk of coronary heart disease and early death [2]. Older adults undergo a natural decline in physical activity due to age-related changes [3], with the sharpest decline in activity in the most senior older adults, at age ≥ 75 years [4]. Current UK guidelines state that older adults should engage in 150 min of moderate-intensity physical activity or 75 min of vigorous-intensity physical activity a week [5]. According to the National Health Service in 2019, in the UK, approximately 40% of adults aged 60–65 years do not meet these guidelines, increasing substantially to 68% in adults aged over 75 years [5]. It should be noted that this statistic is taken from pre-COVID-19 data. Physical inactivity exacerbates the age-related decline in functional performance, health, and quality of life and is linked to mortality [6]. Therefore, it is imperative to encourage older adults to adopt and maintain a physically active lifestyle for their physical health and quality of life.

Not only has physical activity been found to have a benefit to health, fitness [2], and cognition [7], but regular exercise has also been shown to impact mental wellbeing in older adults positively. In this context, it is important to highlight that physical activity and exercise are overlapping but different constructs. Physical activity is defined as any bodily movement produced by skeletal muscles that results in energy expenditure while exercise is a subset of physical activity which is planned, structured, and repetitive and has as a final or an intermediate objective such as the improvement or maintenance of physical fitness [8]. Engaging in physical activity is related to better self-rated health and a decreased likelihood of depressive symptoms in an older population [9].

Football can be used to increase physical activity levels. A number of game formats have been proposed which modify football to benefit the older adult in some way, which include both walking football and recreational football. Walking football has gained interest as a mode of PA for older adults in the UK and more widely [10,11,12] and comprises the game of football but where running is not permitted. Recreational football is a different form of the game of football, but also one where older adults participate [13]. In recreational football, where participants are physically able, running is permitted resulting in a more significant physiological benefit to players [12]. In recreational football, the rules are adapted to minimise the risk to participants. They include not placing the foot on top of the ball and only allowing block tackles (as this reduces risk of falls). Prior studies have shown recreational football for health efficacious in hypertensive, middle-aged males, adults with prostate cancer, and premenopausal women [14]. In older adult participant groups, recreational football has also been found to positively influence health related variables including improving functional capacity [11,13], reducing resting in blood pressure [10], and improving bone mineral density [15].

While understanding such physiological changes is beneficial, it is equally important to examine the experiences of older adults undertaking recreational football. Studies of this nature have only been undertaken in those who have played solely walking football [16]. Exploring the experiences of older adult recreational footballers is a pivotal way to better understand issues around adherence and barriers and facilitators to this mode of physical activity.

One approach to tackle physical inactivity is to understand the behaviour change process to help individuals increase physical activity levels [17]. Many theories have been derived over the years to attempt to explain behaviour change in humans [18,19,20]. The most current evidence-based behaviour change tool is the behaviour change wheel (BCW) and the accompanying COM-B framework [21]. Developed from an umbrella review, the BCW is a standard tool for understanding behaviour. The framework is underpinned by the COM-B framework, which suggests that capability, opportunity, and motivation influences and generates behaviour change [21,22]. Each part of COM can be split further: physical and psychological capability, social and physical opportunity, and reflective and automatic motivation. More information can be found elsewhere [22], but a summary follows. Physical capability involves musculoskeletal functioning, whereas psychological capability involves mental functioning. Physical opportunity relates to the environment and time, while social opportunity regards people and organisations, e.g., social and cultural norms. Finally, reflective motivation necessitates conscious thought processes, such as plans, while automatic motivation entails habitual and drive-related processes, such as habits and desires. Although the premise of the BCW is to inform the design of future interventions, this paper proposes that the framework can be used as a deductive analysis of the data due to the similarities with the theory discovered in the responses. To date, no study has used the BCW and COM-B framework in understanding older adults’ experiences of group-based sports-type interventions.

Although the physiological effects of recreational football on health in older adults have been well documented [23], to date, no study has examined a culmination of the experiences of those older adults who partake in it, the reasoning to stop playing football in the first place, or what changed for those older adults to make them start playing again. Understanding the experiences of this form of activity from participants themselves is an essential first step in facilitating adherence to exercise in older adults. Moreover, learning what stopped adults from playing football and why they are playing now can tell us more about what is necessary to take up football as a sport at this age. Therefore, the aim of this study was to better understand the lived experiences of older adults participating in recreational football for health, using thematic analysis and the BCW framework to analyse the components, including the barriers to and motivators of taking part in a recreational football for health intervention in an older adult population.

This study explored the following research questions:

(1) What stopped older adults from playing football?
(2) Why are older adults playing football now?
(3) How can football continue to be in older adults’ lives in the future?

## 2. Materials and Methods

The following researcher stance and methods used arose from a pragmatic position whereby the research process in the present study recognises that preconceived views on barriers and motivators to playing recreational football would be reflected in participant responses.

### 2.1. Participants

Following ethics approval (Coventry University Ethics committee, P92159) and informed consent, fourteen participants (n = 1 female) aged 67 ± 5.1 years (range: 61–79 years) were recruited from a purposive sample of 25 older adults (comprising 21 males, 4 females) who had taken part in a 12-week, mixed-sex, recreational football intervention run by a UK University. Full details of the recreational football for health intervention are reported elsewhere [13], but in brief, the intervention comprised twice-weekly recreational football sessions comprising a 15 min warm up using a RAMP (raise, activate and mobilise, and potentiate) protocol, followed by a series of six four-minute small-sided games comprising 4 × 4 or 3 × 3 participant numbers with a four-minute rest period between games and a five-minute cool down for a total of 60 min per session. The focus group interviews we report in the current study took part at the end of the 12-week intervention and the number of participants who therefore took part in the focus group interviews represents 56% of the overall population that took part in the intervention itself. All male participants were Caucasian, and the female participant was of African Caribbean ethnic origin. Older adults were recruited based on the World Health Organisation (WHO) criterion of an older adult being an individual aged 60 years or more [24]. Data collection took place pre-COVID-19 and face to face in 2019. Eligibility criteria were no evidence of cardiovascular incident within the past 12 months, no existing or significant past medical history of vascular disease, cancer, diabetes, osteoporosis, or history of falls, no known cognitive impairment, no neuromuscular disorder or injury, and no presence of uncontrolled hypertension. During recruitment, participants completed the International Physical Activity Questionnaire [25] to determine the baseline physical activity levels of the participants. When classified according to participant characteristics, all participants were classed as either moderately or highly physically active. Thus, the responses of participants in this study should be considered in the context of an active population. Participants were purposively split into two focus groups of 7 participants to cater to participants’ preferences on the ideal time of day for them to attend. Groups were in line with published recommendations where 7 participants per focus group are considered appropriate [26]. Participants attended the focus groups after the intervention period had ended; however, ten of the fourteen participants were still playing recreationally. Some participants from the original intervention had dropped out for various reasons, including injury. Therefore, they may not have participated in the present study because they had since disengaged from the project.

### 2.2. Procedure

A semi-structured interview technique was employed as a flexible approach. Topics can be covered in any order, so long as all that was pre-planned is addressed, which allows the interviewer to go with the flow of the participants resulting in a more natural conversation. Following recommendations in the literature, open-ended questions were asked based on six pre-determined questions that were prepared before the focus groups took place [27,28]. The structure of the focus groups was split into three topic areas: prior barriers to taking part, the experience of the intervention, and sustainability of behaviour. The six questions asked are listed below:

1. When it came to playing football regularly, what do you think it needed for you to take part in it?
2. Why did you choose to take part in the project?
3. How did you find the experience of this project?
4. What has changed as a result of the intervention?
5. If we were to offer this again for other people of your age, what changes do you think we need to make to the programme?
6. When it comes to continuing to play football, what do you think it would take for you to continue to play?

Prompts are recommended alongside the planned questions as they build on what was asked before and can provide the researcher with more information [27]. Therefore, prompts were used, when appropriate, to guide the discussion.

To enable open and honest discussion about views on walking and recreational football, someone involved in the project, but not the delivery of the sessions, delivered the focus groups. This person has already built rapport with the individuals during post-session coffee breaks. The researcher was trained to deliver focus groups. Another researcher was present in the focus groups who co-ran the intervention and had built up a strong relationship with the participants during the intervention period. Each focus group lasted approximately an hour as per published recommendations [29]. It is not possible nor good practice to halt a focus group once 60 min has passed if participants are midway through a response or responses. It is for this reason we use the term ‘approximately an hour’ as in some cases the focus groups exceeded this time to enable participants to finish the point they wished to make.

### 2.3. Collection of Responses

**Figure 1:**
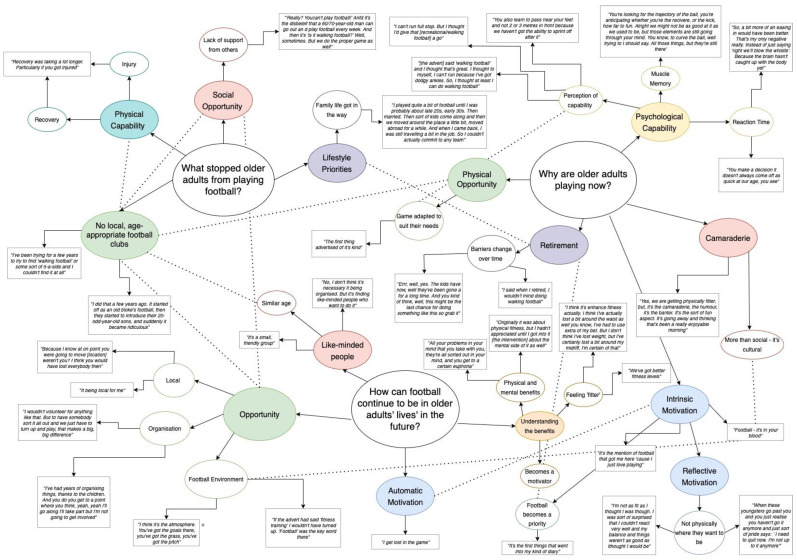
Understanding what stopped participants playing football, what got them playing football, and what is needed for them to continue playing in the future. Bubbles in the same colour indicate the same or similar theme. Dotted lines show links between different themes. In line with the transtheoretical model of behaviour change [20], it is important to note that some themes change from a barrier to a facilitator, depending on the context.

The focus groups were recorded using an Olympus DS-2400 digital voice recorder. The audio was transcribed verbatim by the primary researcher. Following transcription, the transcripts were inductively analysed by the primary researcher. During the coding process, the interviewer was used as a ‘critical friend’ [30] whereby reflection, alternative interpretations, and critical feedback were given, as suggested by Smith and McGannon [31]. A similar discussion process was used during the formation of Figure 1. The inductive approach and the hierarchal organisation of thematic analysis meant that it was an appropriate tool to identify themes in the present study’s data [32].

For qualitative data to be considered trustworthy, the data should have credibility, transferability, dependability, and confirmability [33]. In addition to Braun and Clarke’s thematic analysis stages, Lincoln and Guba’s techniques [33] for establishing trustworthiness were also employed. For example, an audit trail was kept; notes from each researcher were retained and discussed from the interviews and each stage of the analysis process.

### 2.4. Data Analysis

Data was analysed using an inductive–deductive approach. Inductive approaches to qualitative research typically require a theory to be generated from collected data. Meanwhile, a deductive approach to qualitative research will usually utilise a deductivist content analysis whereby data is analysed using a theoretical framework. The inductive-deductive approach is a spectrum, and most researchers will use a blend of the two approaches [34]. Data were inductively coded. When similarities to the BCW framework, a theoretical model, were noticed, deductive analysis was used. The COM-B model (capability, opportunity, and motivation behaviour) is a framework with clear links to specific behavioural techniques which is described in the behaviour change wheel and taxonomoy [22] and provides a synthesis of many established frameworks to explain behaviour [22]. The COM-B model, which sits at the core sources of behaviour in the behaviour change wheel, is recognised by National Institute for health and care excellence as a key framework for understanding and supporting behaviour change NICE [35]. Themes that could be interpreted through the theoretical lens were labelled by theoretical domains, e.g., physical capability, psychological capability, physical opportunity, social opportunity, automatic motivation, and reflective motivation. Items that did not fit into these domains remained labelled inductively.

To clarify how an inductive–deductive approach was employed, inductive coding was used initially in that a data driven approach was employed to enable the identification of factors that may not fit within a theoretical informed interpretation and thus open coding was used to present the meaning communicated from the participants [36]. Once codes and themes were identified, then these were examined through the theoretical lens of the COMB-B model to examine if the theory could inform the data interpretation. More recently, it has been understood that coding and analysis rarely falls into either deductive and inductive approaches and that in reality it uses a combination of both approaches [36,37,38]. It is further argued that it is not possible to conduct exclusive deductive analysis as an appreciation for the relationship between the data is needed. Furthermore, it is argued that exclusive inductive analysis is also not possible as researchers operate from a criterion when they are addressing whether to code items that address the research question [39].

## 3. Results

A summary of the questions, themes, and supporting quotes is displayed in Figure 1 and is described below.

### 3.1. What Stopped Older Adults from Playing Football?

The integration between physical capability, social opportunity, and lifestyles priorities were the key reasons to stop playing football.

Considering physical capability, older adults described how they reached an age where they physically could not keep up with the other players anymore, it was taking too long to recover between training sessions, they were getting injured or felt that they were more likely to get injured, and therefore stopped playing for fear of sustaining an injury or because they were advised by the doctor to stop.

*‘Recovery was taking a lot longer. Particularly if you got injured.’*

(Participant 3)

Participants also reported a lack of support from others. There appears to be a stereotype and misconception that older adults cannot play football because of their age.

*“‘Really? You can’t play football!’ And it’s the disbelief that a 60/70-year-old man can go out and play football every week. And then it’s ‘Is it walking football?’ Well, sometimes. But we do the proper game as well.”*

(Participant 3)

This was further impacted by the lack of age-appropriate football clubs (physical opportunity). They explained how there was nothing available that was appropriate to their age group and how they struggled to find information on recreational football for older adults. They agreed that before the intervention, they wanted to play football, but there was nothing available to them. Some mentioned that they had tried other football groups for people over the age of 50, but they could not keep up with them and got frustrated when other players brought people under the age of 50 along to play. A lack of local, age-appropriate football clubs links social opportunity with physical capability.

*‘I did that a few years ago. It started off as an old bloke’s football, then they started to introduce their 20-odd-year-old sons, and suddenly it became ridiculous.’*

(Participant 14)

*“I’ve been trying for a few years to try to find ‘walking football’ or some sort of 5-a-side and I couldn’t find it at all.”*

(Participant 4)

Alongside this, participants’ lifestyle responsibilities were changing. While the majority reported that they played football in their youth (e.g., at school, during their early 20s, etc.), they also reported how their responsibilities and priorities had changed due to their marriage, jobs, and children, and they no longer had the time to play football.

*‘I played quite a bit of football until I was probably late 20s, early 30s. Then married. Then sort of kids come along and then we moved around the place a little bit, moved abroad for a while. And then when I came back, I was still travelling a bit in the job. So I couldn’t actually commit to any team.’*

(Participant 4)

### 3.2. Why Are Older Adults Playing Now?

The Amalgamation of Camaraderie, Retirement, Intrinsic Motivation, and Psychological Capability Were the Main Reasons Why the Participants Were Playing Football Now.

A common motivator to resume playing football was a lifestyle change. Most of the participants were retired or semi-retired and no longer had work commitments, and their children had grown up and left home. Therefore, they had more time to allocate to other activities, such as walking or recreational football. The motivation to take part has shifted since the participants were younger. Playing football has now become a priority for them, which might only have been the case with the change in lifestyle that naturally comes with age. It is important to note that lifestyle went from a barrier to a facilitator.

*‘Errr, well, yes. The kids have now, well they’ve been gone for a long time. And you kind of think, well, this might be the last chance of doing something like this so grab it.’*

(Participant 3)

*‘I said when I retired, I wouldn’t mind doing walking football or whatever. The trouble was trying to find it.’*

(Participant 9)

Camaraderie appeared to be a prominent reason why participants were playing football now. Many participants enjoyed socialising during the intervention, implying that football has its own kind of social culture. This is perhaps something that is unique to football.

*‘Yes, we are getting physically fitter, but, it’s the camaraderie, the humour, it’s the banter. It’s the sort of fun aspect. It’s going away and thinking that’s been a really enjoyable morning.’*

(Participant 19)

A recurrent theme in the focus groups was a sense amongst interviewees that they wanted to play football because it was fun and enjoyable.

*‘It’s become the highlight of my week.’*

(Participant 14)

*‘It’s been very enjoyable, incredibly enjoyable.’*

(Participant 2)

Participants suggested that they were intrinsically motivated to play football. There appears to be an instinctive drive from within that motivates the participants to play. There was a strong sense that football was of cultural and emotional importance to the participants. Many outlined that their reason for taking part was simply because it was football, suggesting that there was something about the game itself that was motivating.

*‘Football—it’s in your blood.’*

(Participant 16)

*‘It’s the mention of football that got me here ‘cause I just love playing.’*

(Participant 6)

*“It’s the first thing that kind of went in my diary.”*

(Participant 11)

There is also an element of reflective motivation in the participants. They seem to express that they are not physically where they want to be, which perhaps becomes a motivator for them to continue playing.

*‘When these youngsters go past you and you just realise you haven’t got it anymore and just sort of pride says: “I need to quit now. I’m not up to it anymore.”’*

(Participant 19)

*‘I’m not as fit as I thought I was though. I was sort of surprised that I couldn’t react very well and my balance and things weren’t as good as I thought I would be.’*

(Participant 10)

There was a strong consensus that the participants had a perception that their cognitive ability had declined from what it had been when they were younger. Participants suggested that their reaction time was slower to stimuli when playing football. There appears to be a relationship between the mind and the body.

*‘So, a bit more of an easing in would have been better. That’s my only negative really. Instead of just saying ‘right we’ll blow the whistle’. Because the brain hasn’t caught up with the body yet.’*

(Participant 19)

*‘You make a decision it doesn’t always come off as quick at our age, you see.’*

(Participant 14)

The idea of ‘muscle memory’ was raised, with participants expressing that it was as if their brains could remember the movement patterns from when they played in their youth. The psychological capability of the players has practical implications and should be considered by coaches when delivering football sessions to older adults.

*‘You’re looking for the trajectory of the ball, you’re anticipating whether you’re the receiver, or the kick, how far to run. Alright we might not be as good at it as we used to be but those elements are still going through your mind. You know, to curve the ball, well trying to I should say. All those things, but they’re still there.’*

(Participant 14)

Moreover, participants had a perception that they did not have the physical capability to play ‘normal’ football. This links to physical opportunity, whereby the game has been adapted to be easier to play considering the prerequisites of an older adult.

*‘[the advert] said “walking football” and I thought that’s great. I thought to myself, I can’t run because I’ve got dodgy ankles. So, I thought at least I can do walking football.’*

(Participant 11)

*‘But you also learn to pass near your feet and not 2 or 3 m in front because we haven’t got the ability to sprint off after it.’*

(Participant 19)

*‘I can’t run full stop. But I thought I’d give that [recreational football] a go.’*

(Participant 11)

The adapted version of the sport, walking football, was a key driver for making the change from not playing to playing again. Adapting the game to suit the needs of older adults was pivotal to making the change to start playing again. This links to the perception of their own capability, whereby participants felt that they could not keep up with the demands of the normal version of the game.

Walking football provides the physical opportunity to address the barriers; no local age-appropriate clubs and the misconception that older adults can play football. Having like-minded people (social opportunity) and the adapted rules of the game (physical capability) can overcome these barriers.

*‘You also thought you could do more than you could do as well.’*

(Participant 9)

*‘The first thing advertised of its kind.’*

(Participant 10)

### 3.3. How Can Football Continue to Be in Older Adults’ Lives in the Future?

Finally, a Combination of Like-Minded People, Facilitators, Understanding the Benefits, Being Intrinsically Motivated, and Having a Group of Local, Organised People, Were Considered as Fundamental Staples for Football to Continue to Be in Their Lives.

Having like-minded people was considered crucial to continue playing football. Most participants agreed that having a group of friendly, like-minded people was necessary to continue playing.

*‘No, I don’t think it’s necessary about it being organised. But it’s finding like-minded people who want to do it.’*

(Participant 9)

*‘It is quite a small, friendly group, which helps.’*

(Participant 1)

Participants were able to reflect on what was good for them as a result of taking part in the program. Many had the perspective that playing football had benefited their mental well-being. Several participants revealed that they use exercise as a stress reliever.

*‘Originally it was about physical fitness, but I hadn’t appreciated until I got into it [the intervention] about the mental side of it as well.’*

(Participant 19)

*‘To me, exercise has always been a big escape valve.’*

(Participant 16)

As well as mental wellbeing, physical wellbeing was also reflected as a benefit of the program. Some interviewees felt that they needed to get fitter as they had become lazy during retirement while others considered that they wanted to get fitter to be able to keep up with their grandchildren. Therefore, there was a self-perceived benefit to physical fitness from playing football that became a motivation to continue playing.

*‘We’ve got better fitness levels.’*

(Participant 9)

*‘I think its enhanced fitness actually. I think I’ve actually lost a bit around the waist as well you know I’ve had to use extra of my belt. But I don’t think I’ve lost weight, but I’ve certainly lost a bit around my midriff, I’m certain of that.’*

(Participant 14)

Another key theme when considering motivations to continue playing was the idea of automatic motivation. Playing football appears to provoke drive states and emotional responses from the participants, thus suggesting that participants are automatically motivated to continue playing because of their love for the game.

*‘I get lost in the game.’*

(Participant 19)

Furthermore, there was a strong consensus that for the participants to continue to play football, the sessions need to be organised by someone else. Many felt that they had spent their lives organising, and now they were retired; they did not want that responsibility anymore. There seemed to be some dissonance between the participants when it came to discussing what was needed going forward. While some participants believed a facilitator was necessary, others believed that football could continue without a facilitator so long as there was a group of people who all loved football.

*‘I’ve had years of organizing things, thanks to the children. And you do you get to a point where you think, yeah, yeah I’ll go along I’ll take part but I’m not going to get involved.’*

(Participant 1)

*‘I wouldn’t volunteer for anything like that. But to have somebody sort it all out and we just have to turn up and play, that makes a big, big difference.’*

(Participant 16)

Many believed that keeping it local would help them continue to play. This theme came up, for example, in discussions of moving location.

*‘Because I know at one point you were going to move weren’t you? I think you would have lost everybody then.’*

(Participant 23)

*‘It being local for me.’*

(Participant 14)

Emulating the football environment and creating a traditional football culture was identified as necessary by the participants. To play football, certain elements that they love from the game must be apparent, e.g., a good playing surface and proper goals, which were unfortunately unavailable during the initial intervention. The opportunity to play with other teams was also mentioned; therefore, the competitive element of football is a critical motivator which could encourage older adults to continue playing football.

*‘I think it’s the atmosphere. You’ve got the goals there, you’ve got the grass, you’ve got the pitch.’*

(Participant 10)

Many compared playing football to going to the gym or going for a run. While the minority of participants mentioned that they enjoyed the gym or running, most agreed that those activities were not enjoyable or motivating. Being in the football environment appears to be key here, which links back to the idea that football is intrinsically motivating. There is something about football, compared to other exercises, that is motivating in itself.

*‘If it [the advert] has said “fitness training” I wouldn’t have turned up. “Football” was the keyword there.’*

(Participant 14)

## 4. Discussion

The current study presents qualitative data exploring the lived experiences of older adults undertaking walking and recreational football. It is the first study to explore the experiences of those who have played walking and recreational football, while simultaneously exploring what stopped older adults playing football to begin with, and what is necessary to continue playing in the future. The use of the BCW in the present study to identify elements of capability, opportunity, and motivation for playing walking and recreational football in an older adult population, is, however, a unique feature, which is crucial in enabling public health practitioners, community sports, and local government providers to construct effective, future interventions. The main finding from this study was that football needs to be adapted to suit the needs of older adults, whilst also being available in the local community. The number of specific walking and recreational football clubs for adults aged over 60 years old needs to be increased to help address the physical inactivity of the older adult population. Another novel finding of the present study was that priority to play football changes over time. Key life occurrences, such as work and children, de-prioritise football in people’s lives. Once these are no longer prevalent, e.g., when retired, football is bumped up the priority list. Additionally, there is something about football itself that is intrinsically motivating participants to play. Understanding that priorities change over time but also that football motivates certain players from within, those that have football in their blood, can help to encourage and maintain adherence to a physically active lifestyle. Finally, the present study found that having fun was an important motivator for participating in the program; the fun comes from a multitude of elements including the camaraderie of the group and getting lost in the game.

Similar themes have been identified in a recent study looking at the sustainability of walking football in older adults. Interviews showed that participants found football enjoyable, sociable, and made the players feel more confident and that they believed football had improved their health and wellbeing [12]. Moreover, an ethnographic study where the researcher immersed themselves in a walking football club found that health, wellbeing, enjoyment, and inclusion were the main reasons for taking part [16]. The present study differs from the work of Loadman [16] as it reflects the views of a wider group of individuals engaged in the sport. In contrast, the aforementioned study employed ethnography, thus requiring experiences to be filtered through the understanding of one individual. A key differentiating factor of the present study from prior relevant work is the focus on recreational football rather than walking football. The relevance of walking vs recreational football is important as the physical demands of recreational football are greater. This is particularly the case as, although walking football is regarded as an engaging and social activity for older adults, the direct health and cognitive benefits are considered minimal due to the low exercise intensity [12]. Therefore, it is important to understand the different experiences of older adult recreational footballers as the physiological benefit is more significant than in walking football.

### 4.1. Barriers

Despite the benefits of regular physical activity, qualitative studies have identified many barriers to adopting a physically active lifestyle for older adults: time constraints, physical and mental health, risk of injury, safety concerns, and environmental and organisational factors [40]. Rasinaho et al. asked participants with mobility limitations about the motives and barriers to exercise [41]. They found that poor health, fear of negative experiences, lack of company, and an unsuitable environment were the most reported barriers to engaging in physical activity. However, few studies have examined the barriers to playing football in an older population, and no study to date has discussed this in the context of recreational football.

The barriers identified in the present study were similar to those identified in other focus groups of exercising older adults: physical decline, injury, cognitive decline, and lack of local facilities/clubs [40]. However, previously unreported barriers were identified (stereotypes or misconceptions). It is important to note that the prior studies were a mixture of general studies on physical activity, including community-dwelling active and inactive older adults, and were not recreational football studies. Therefore, the present study adds new findings on the experiences, barriers, and motivators to the recreational football for health literature.

### 4.2. Facilitators

A key theme from the analysis was the need to understand the benefits of recreational football as a motivation to continue playing. Participants used football as a stress reliever and believed that playing football helped improve their mental wellbeing. Playing regular football has been reported in the literature as a supplementary treatment for several mental health illnesses. For example, football has been used as an additional treatment for patients suffering from mild depression [42] and schizophrenia [43]. The idea that football is good for mental wellbeing is supported by the NICE guidelines, which suggest that individuals expressing symptoms of depression should engage in group physical activity at least three times a week [44]. Interviews from those who took part in a mental health football project revealed that participants perceived the benefits of taking part were improved social relationships, recovery, and improved physical and mental wellbeing. Similarly, maintaining fitness and improving mental wellbeing were all identified as motivators to continue playing recreational football in the present study.

Although the present study implies that playing football can benefit mental wellbeing in older adults, this paper did not look at mental health. Although different from mental health, mental wellbeing has been posited as a component of mental health [45]. Poor mental wellbeing has been linked to incidences of mental health [45]; therefore, good mental wellbeing can improve mental health [45]. However, it is essential to note that the occurrence of any mental health illness was not measured in this study. Therefore, the findings from the present study do not directly reflect any incidences of mental health in this population. Currently, it can only be inferred from these focus groups that recreational football can improve the mental wellbeing of older adults.

Another reason for taking part in the intervention was because it was a team sport and the camaraderie that comes with that. Many participants implied that they did not like exercising at a gym because they found it lonely. The literature suggests that being physically active in a group setting is good for mental wellbeing [46]. Other physical activity research in older adults has indicated that improving health and socialising are key motivators for engagement in physical activity [46]. Thus, the present study supports previous findings in the literature that social opportunity is key for changing and maintaining positive behaviours for being physically active. Social connections appear to be an important part of the football culture for older adults.

Moreover, another study identified three health and wellbeing themes from their qualitative data after a football for mental health intervention in males over the age of 50. When reporting on their personal experience of the intervention, participants reported three key impacts of the programme. Firstly, the intervention gave them the opportunity for positive social interaction. Secondly, the intervention enabled the group to motivate each other to improve their health. Finally, playing football gave participants a new lease on life [47]. These findings imply that playing football has more than just an effect on mental wellbeing in an older population, thus supporting the conclusions of the present study that football can positively impact social opportunity.

In addition, Reddy et al. found after playing walking football for an hour a week, over three months, older adult participants reported that playing was an enjoyable and sociable experience [12]. Similar views on enjoyment and positive social experiences were expressed in the present study. Football might be a noteworthy opportunity for social interaction in older adults. The difference with the present study was that the participants played both walking and recreational football. Thus, evidence has shown that both walking and recreational football can have a positive social benefit for the participants. The intensity of the football sessions does not appear to change the social impact.

From the analysis, participants showed automatic motivation to play football. For example, football evokes an emotional response for most participants and engenders desires and drive responses. For example, there appears to be a desire to play football and playing football gives the participants pleasure. Furthermore, football appears to be of cultural significance to the participants, stemming from the idea that it is in their blood. Therefore, it can be inferred that football seems to play a central role in the everyday lives of many of the participants. These ideas are congruent with the themes derived from the focus groups in the present study. Because the intervention was centered on football, it served as a mooring point, thus enabling the participants to enjoy the physical activity more so than other forms of exercise, such as the gym (which many expressed a dislike for during the focus groups). Moreover, the participants also felt a sense of belonging as they experienced positive social interactions because they were a part of the football programme. Therefore, the significance of the program being football is perhaps something that should be further explored. The extent to which the programme’s benefits were directly a result of it being football, or if it was just physical activity in general, should be studied to help distinguish the benefits of different modes of physical activity for different people.

### 4.3. Sustaining Behaviour Change through Recreational Football

Evidence suggests that behaviour change techniques for physical inactivity used on the youth are ineffective in older adults [17], most likely due to age-related physiological differences, as older adults experience a decline in physiological capability. Therefore, it is important to adapt sports to suit the needs of an older population. Without adjusting the sport, older adults will not engage, and a change in sedentary behaviour will not occur. This is confirmed in the present study, where the participants unanimously agreed that a barrier, motivator, and requirement for future adherence was whether the sport was adapted to suit their cognitive and functional needs as an older person.

Thematic analysis of the transcripts revealed that, for the participants to continue to play, sessions need to be run by a facilitator. Some stated that organising to play themselves would not be feasible as they feel they do not want to organise such activities now that they have reached a certain age. Parnell’s ‘Extra Time’ program is further evidence of the need to have a facilitator [48]. The ‘Extra Time’ program used professional football clubs to reach older adults to engage them in physical activity. Interviews with participants revealed that the program was effective in helping engage older adults in physical and social activities [48]. The program from the present study facilitated an increased opportunity to play football, which perhaps without the program, the chance to play football again would not have occurred. Moreover, the participants in this study revealed that even if they had to pay to take part, that would not stop them from participating. Finally, the location was another key theme identified; football sessions need to be close to home. The present results emphasise the need for football to be an accessible activity for many communities to be able to engage with it.

### 4.4. Strengths and Limitations

This is the first study that has explored barriers to playing football, the experiences of playing now, and what is needed to continue playing football as an older adult. Another unique aspect of the present study is the process of behaviour change. The analysis identified incidences of the theoretical framework: the BCW and COM-B model. Therefore, a significant strength of the current study was the detail of information gathered on how older adults feel about participating in a recreational football program, combined with part of the analysis having theoretical underpinning. Understanding how participants’ experiences might change in recreational football is essential in developing sustainable, safe, and effective football-based physical activity interventions for older adults. The present study provides this information.

We acknowledge that the results of this study reflect the perceptions of a relatively small number of participants who were predominantly male and Caucasian. The participants from the present study were self-selected, which minimises bias. This could be viewed as a potential source of bias whereby participants decided to participate in the focus groups because they had a predominantly positive experience during the intervention. Moreover, the gender balance of participants is skewed. Nonetheless, this was similar to the overall ratio of males to females engaging in the recreational football programme from which the participants were recruited. The female participant expressed similar views to the males in the present study suggesting that the uneven ratio may not be a meaningful issue when interpreting the results of this study.

Although focus groups allow respondents to build on each other’s thoughts and enable the researcher to develop a rich understanding of the collective responses to this kind of intervention, it is possible that some participants may not have felt able to speak their minds. However, the focus groups were conducted according to guidelines [36,37,38], thus ensuring everyone could contribute. To date, the COM-B framework and the BCW [21,22] have seldom been used in older adult research. However, the use of these tools adds more depth to the exploration of the experiences of the participants in the recreational football programme. The COM-B framework tells us what drives behaviour; thus, the present study not only looks at the perceptions of the participants but uses a tool to add context and identify what needs to change to improve engagement with football in the older adult population.

## 5. Conclusions

This study is the first to explore the experiences of older adults in a walking and recreational football intervention, while also understanding what barriers stopped them from playing prior to the intervention and asking what is needed to continue playing. The key finding here is that football was in the participants’ blood (intrinsic motivation) but they reached an age where they were getting injured, taking longer to recover (physical capability), and there were no local age-appropriate clubs they could transition to. This coincided with a time in their life when priorities were changing, e.g., married life and family responsibilities. As an older adult who is retired, the love for the game is still there and walking and recreational football provides the opportunity to play the game adapted to physical capability, with like-minded people.

The results from the present study have a broader application. For example, for older adults to engage in recreational football, a coach or practitioner must facilitate the programme, and the location of the programme must be accessible and local for them to want to take part. Therefore, the results could be used by coaches, practitioners, public health providers, and local governments to increase adherence and uptake of recreational football, which could, in turn, increase physical activity levels in older adults.

## 6. Application

Sports leaders and health promoters can utilise the findings presented. They need to ensure opportunities for activities are provided locally with like-minded individuals and that football is adapted to suit the needs of older adults, e.g., providing the opportunity for walking football for those who feel or do not have the capability to run.

Moreover, the participants’ responses allude to the idea of physical literacy. Those that have good experiences with football early on in life develop the ability to play lifelong sport. This should be a key consideration to coaches and practitioners, who should make an effort to provide as many positive experiences when coaching children, as early experiences could be key to returning to football in later life.

## Data Availability

Not applicable.

## References

[1] World Health Organisation (WHO) (2018). NCDs Physical Inactivity: A Global Public Health Problem.

[2] Chodzko-Zajko W.J., Proctor D.N., Fiatarone Singh M.A., Minson C.T., Nigg C.R., Salem G.J., Skinner J.S., American College of Sports Medicine (2009). American College of Sports Medicine position stand. Exercise and physical activity for older adults. Med. Sci. Sports Exerc..

[3] Lohne-Seiler H., Hansen B.H., Kolle E., Anderssen S.A. (2014). Accelerometer-determined physical activity and self-reported health in a population of older adults (65–85 years): A cross-sectional study. BMC Public Health.

[4] (2019). Physical Activity Guidelines: UK Chief Medical Officers’ Report—GOV.UK. https://www.gov.uk/government/publications/physical-activity-guidelines-uk-chief-medical-officers-report.

[5] NHS England (2019). NHS England Practical Guide to Healthy Ageing.

[6] Ding D., Lawson K.D., Kolbe-Alexander T.L., Finkelstein E.A., Katzmarzyk P.T., van Mechelen W., Pratt M., Lancet Physical Activity Series 2 Executive Committee (2016). The economic burden of physical inactivity: A global analysis of major non-communicable diseases. Lancet.

[7] Geda Y.E., Roberts R.O., Knopman D.S., Christianson T.J., Pankratz V.S., Ivnik R.J., Boeve B.F., Tangalos E.G., Petersen R.C., Rocca W.A. (2010). Physical exercise, aging, and mild cognitive impairment: A population-based study. Arch. Neurol..

[8] Caspersen C.J., Powell K.E., Christenson G.M. (1985). Physical activity, exercise, and physical fitness: Definitions and distinctions for health-related research. Public Health Rep..

[9] Ruuskanen J.M., Ruoppila I. (1995). Physical Activity and Psychological Well-being among People Aged 65 to 84 Years. Age Ageing.

[10] Reddy P., Dias I., Holland C., Campbell N., Nagar I., Connolly L., Krustrup P., Hubball H. (2017). Walking football as sustainable exercise for older adults—A pilot investigation. Eur. J. Sport Sci..

[11] Sundstrup E., Jakobsen M.D., Andersen L.L., Andersen T.R., Randers M.B., Helge J.W., Suetta C., Schmidt J.F., Bangsbo J., Krustrup P. (2016). Positive effects of 1-year football and strength training on mechanical muscle function and functional capacity in elderly men. Eur. J. Appl. Physiol..

[12] Arnold J.T., Bruce-Low S., Sammut L. (2015). The impact of 12 weeks walking football on health and fitness in males over 50 years of age. BMJ Open Sport Exerc. Med..

[13] Duncan M.J., Mowle S., Noon M., Eyre E., Clarke N.D., Hill M., Tallis J., Julin M. (2022). The Effect of 12-Weeks Recreational Football (Soccer) for Health Intervention on Functional Movement in Older Adults. Int. J. Environ. Res. Public Health.

[14] Bruun D.M., Bjerre E.J., Krustrup P., Brasso K., Johansen C., Rørth M., Midtgaard J. (2014). Community-Based Recreational Football: A Novel Approach to Promote Physical Activity and Quality of Life in Prostate Cancer Survivors. Int. J. Environ. Res. Public Health.

[15] Helge E.W., Andersen T.R., Schmidt J.F., Jørgensen N.R., Hornstrup T., Krustrup P., Bangsbo J. (2014). Recreational football improves bone mineral density and bone turnover marker profile in elderly men. Scand. J. Med. Sci. Sports.

[16] Loadman A. (2019). “He’s running, Ref!” An ethnogrpahic study of walking football. Soccer Soc..

[17] French D.P., Olander E., Chisholm A., Mc Sharry J. (2014). Which Behaviour Change Techniques Are Most Effective at Increasing Older Adults’ Self-Efficacy and Physical Activity Behaviour? A Systematic Review. Ann. Behav. Med..

[18] Hochbaun G. (1958). Public Participaiton in Medical Screening Programs: A Socio-Psychological Study.

[19] Ajzen I. (2011). The theory of planned behaviour: Reactions and reflections. Psychol. Health.

[20] Prochaska J., Norcorss J. (1990). Systems of Psychotherapy: A Trasntheoretical Analysis.

[21] Michie S., Wood C.E., Johnston M., Abraham C., Francis J.J., Hardeman W. (2015). Behaviour change techniques: The development and evaluation of a taxonomic method for reporting and describing behaviour change interventions (a suite of five studies involving consensus methods, randomised controlled trials and analysis of qualitative data). Health Technol. Assess..

[22] Michie S., Van Stralen M.M., West R. (2011). The behaviour change wheel: A new method for characterising and designing behaviour change interventions. Implement. Sci..

[23] Andersen T.R., Schmidt J.F., Nielsen J.J., Randers M.B., Sundstrup E., Jakobsen M.D., Andersen L.L., Suetta C., Aagaard P., Bangsbo J. (2014). Effect of football or strength training on functional ability and physical performance in untrained old men. Scand. J. Med. Sci. Sports.

[24] World Health Organization Ageing. https://www.who.int/health-topics/ageing#tab=tab_1.

[25] IPAQ (2005). Guidelines for Data Processing Analysis of the International Physical Activity Questionnaire (IPAQ): Short and Long Forms. http://www.ipaq.ki.se/.

[26] McIntosh M.J., Morse J.M. (2015). Situating and Constructing Diversity in Semi-Structured Interviews. Glob. Qual. Nurs. Res..

[27] Gill P., Stewart K., Treasure E., Chadwick B. (2008). Methods of data collection in qualitative research: Interviews and focus groups. Br. Dent. J..

[28] Krueger R.A., Casey M.A. (2000). Focus Groups: A Practical Guide for Applied Research.

[29] Braun V., Clarke V. (2006). Using Thematic Analysis in Psychology. Qual Res. Psychol..

[30] Smith B., Sparkes A.C. (2006). Narrative enquiry in psychology: Exploring the thensions within. Qual. Res. Psychol..

[31] Smith B., McGannon K.R. (2018). Developing rigour in qualitative research: Problems and opportunities within sport and exercise psychology. Int. Rev. Sport Exerc. Psychol..

[32] Braun V., Clarke V., Weate P., Smith B., Sparkes A.C. (2016). Using thematic analysis in sport and exercise research. Routledge Handbook of Qualitative Research in Sport and Exercise.

[33] Lincoln Y.S., Guba E.G. (1985). Naturalistic Inquiry.

[34] Graebner M., Martin J.A., Roundy P. (2012). Qualitative data: Cooking without a recipe. Strat. Organ..

[35] NICE (2014). Behaviour Change: Individual Approaches Public Health Guideline [PH49].

[36] Braun V., Clarke V. (2013). Successful Qualitative Research: A Practical Guide for Beginners.

[37] Braun V., Clarke V. (2019). Reflecting on reflexive thematic analysis. Qual. Res. Sport Exerc. Health.

[38] Braun V., Clarke V. (2021). One size fits all? What counts as quality practice in (reflexive) thematic analysis?. Qual. Res. Psychol..

[39] Bingham A.J., Witkowsky P., Vanover C., Mihas P., Saldaña J. (2022). Deductive and inductive approaches to qualitative data analysis. Analyzing and Interpreting Qualitative Data: After the Interview.

[40] Lees F.D., Clark P.G., Nigg C.R., Newman P. (2005). Barriers to Exercise Behavior among Older Adults: A Focus-Group Study. J. Aging Phys. Act..

[41] Rasinaho M., Hirvensalo M., Leinonen R., Lintunen T., Rantanen T. (2007). Motives for and Barriers to Physical Activity among Older Adults with Mobility Limitations. J. Aging Phys. Act..

[42] Lamont E., Harris J., McDonald G., Kerin T., Dickens G. (2017). Qualitative investigation of the role of collaborative football and walking football groups in mental health recovery. Ment. Health Phys. Act..

[43] Battaglia G., Alesi M., Inguglia M., Roccella M., Caramazza G., Bellafiore M., Palma A. (2013). Soccer practice as an add-on treatment in the management of individuals with a diagnosis of schizophrenia. Neuropsychiatr. Dis. Treat..

[44] NICE (2009). Depression in Adults: Recognition and Management Guidance.

[45] De Cates A., Stranges S., Blake A., Weich S. (2015). Mental well-being: An important outcome for mental health services?. Br. J. Psychiatry.

[46] Jones D.B., Richeson N.E., Croteau K.A., Farmer B.C. (2009). Focus groups to explore the perceptions of older adults on a pedometer-based intervention. Res. Q. Exerc. Sport.

[47] McKeown M., Roy A., Spandler H. (2015). ‘You’ll never walk alone’: Supportive social relations in a football and mental health project. Int. J. Ment. Health Nurs..

[48] Parnell D., Pringle A., McKenna J., Zwolinsky S., Rutherford Z., Hargreaves J., Trotter L., Rigby M., Richardson D. (2015). Reaching older people with PA delivered in football clubs: The reach, adoption and implementation characteristics of the Extra Time Programme. BMC Public Health.

